# Exploring the association between anhedonia and nicotine dependence: A study among female undergraduate students in Saudi Arabia

**DOI:** 10.18332/tid/203551

**Published:** 2025-04-30

**Authors:** Mai B. Alwesmi, Sana Hawamdeh, Sondus F. Alotaibi, May A. Alfohaid, Futun M. Alharbi, Nourah A. Alghamdi, Jumanah K. Alghamdi, Fai A. Aseeri, Raghad A. Alqhatani, Adam Saleh

**Affiliations:** 1 Department of Medical-Surgical Nursing, College of Nursing, Princess Nourah bint Abdulrahman University, Riyadh, Saudi Arabia; 2 School of Nursing, University of Pennsylvania, Philadelphia, United States; 3 College of Nursing, Princess Nourah bint Abdulrahman University, Riyadh, Saudi Arabia; 4 Faculty of Medicine, Near East University, Nicosia, Cyprus

**Keywords:** anhedonia, nicotine, smoking, e-cigarettes, vape

## Abstract

**INTRODUCTION:**

Nicotine dependence and its psychological foundations, including anhedonia, are major public health issues, especially among young adults. There is a dearth of knowledge regarding nicotine dependence and anhedonia especially among female young adults. Thus, this study aimed to investigate the associations between anhedonia and nicotine dependence among female undergraduate students.

**METHODS:**

A cross-sectional study was conducted in March 2024, among 449 female undergraduate students, in Saudi Arabia. Data were collected using the Fagerström test for nicotine dependence (FTND) and the Snaith-Hamilton Pleasure Scale (SHAPS).

**RESULTS:**

A total of 449 female undergraduate students participated in the study. The majority were aged 18–20 years (62.8%). The study found that 11.4% of participants reported nicotine use, primarily e-cigarettes (66.7%). Nicotine users reported higher parental [45.1% vs 26.4%, χ^2^(1)=7.770, p=0.005] and sibling nicotine use [52.9% vs 30.7%, χ^2^(1)=17.992, p=0.001]. Mental health conditions were more prevalent in nicotine users [39.2% vs 15.1%, χ^2^(1)=17.992, p<0.001]. Logistic regression identified mental health conditions (OR=4.44, p<0.001), sibling nicotine use (OR=2.37, p=0.006), and parental nicotine use (OR=2.27, p=0.01) as key predictors of nicotine use. Anhedonia was present in 19.8% of participants, associated with mental health conditions [38.2% vs 12.8%, χ^2^(1)=31.501, p<0.001], nicotine use [27% vs 7.5%, χ^2^(1)=8.309, p=0.005] and sibling nicotine use [46.1% vs 30%, χ^2^(1)=26.857, p<0.001]. Mental health conditions (OR=3.47, p<0.001) and nicotine use (OR=3.34, p<0.001) strongly predicted anhedonia.

**CONCLUSIONS:**

The study's results support the notion that psychological discomfort influences nicotine use, demonstrating a substantial association between anhedonia and nicotine use. Given the influence of familial nicotine use, there is an immediate need for targeted interventions that address both social and psychological aspects.

## INTRODUCTION

Nicotine dependence and its psychological foundations, including anhedonia, are major public health issues, especially among young adults^1^. The transition to university life is a pivotal phase during which smoking behaviors may develop or escalate, shaped by many social, psychological, and familial influences^2^. Anhedonia, which is defined as the inability to experience pleasure, is a major feature of mood disorders such as depression. It has also been related to substance use disorders, particularly nicotine dependence, in a growing number of cases^3^. Research has clarified the complex association between anhedonia and nicotine dependence, suggesting that individuals with heightened anhedonia are more likely to use nicotine as a self-medication approach^3-5^. This usage may provide transient mood enhancement, but it ultimately establishes a cycle of reliance in which nicotine use and anhedonia mutually reinforce one another^6^.

The association between anhedonia and nicotine dependence is tied to the brain’s mesolimbic dopamine system, especially the ventral tegmental area (VTA) and nucleus accumbens (NAc), which regulate reward and motivation. When nicotine enters the system, it triggers dopamine release in these areas, briefly easing anhedonia. However, prolonged nicotine exposure leads to neuroadaptations that impair dopamine signaling, ultimately reducing the brain’s ability to experience pleasure naturally^7,8^. This disruption contributes to both nicotine dependence and withdrawal challenges, as individuals with heightened anhedonia experience more severe withdrawal symptoms, including acute cravings and psychological distress, leading to higher relapse rates9,10.

Anhedonia also plays a role in the initiation and maintenance of smoking behaviors. Leventhal et al.^11^, revealed that anhedonia significantly predicts smoking onset, especially in teenagers, while Kang and Malvaso^12^ found that this relationship also applies to e-cigarette use, emphasizing anhedonia’s substantial impact on nicotine use patterns. People experiencing anhedonia are more likely to use nicotine as a coping mechanism, thereby increasing their susceptibility to nicotine dependence^12^. Once dependence develops, nicotine’s short-term mood-enhancing effects sustain the addiction, exacerbating anhedonia over time^8,13^.

Individuals with reduced capacity for pleasure find it challenging to sustain abstinence, underscoring the necessity for customized therapy that addresses both nicotine dependence and the underlying anhedonic symptoms^14^. Recent studies have prompted scientists to investigate the possibility of developing interventions for this population. A previous study proposed that smoking cessation programs incorporating behavioral interventions to improve reward sensitivity may be more efficacious for persons suffering from anhedonia^9^. This technique seeks to rectify deficiencies in the brain’s reward system by providing alternative sources of gratification, hence diminishing nicotine dependence. This corresponds with prior research which has highlighted that anhedonia’s influence on reward processing could result in addictive behaviors, such as nicotine use, and should be incorporated into treatment approaches^15^.

The link between anhedonia and nicotine dependence extends beyond traditional smoking. Previous studies have elucidated the correlation between anhedonia and e-cigarette use, demonstrating that individuals with anhedonia exhibited a higher propensity for regular e-cigarette usage, mirroring the dependence patterns seen with conventional cigarettes^6^. This highlights the substantial effects of anhedonia in nicotine dependence, which can be caused by inhaling nicotine through a variety of different routes.

Research on young adults has revealed the prevalence of nicotine use among college students. Previous research findings illuminate the demographic and behavioral factors contributing to the development of dependence, highlighting the influence of age, peer influence, stress, and accessibility on nicotine use^16-18^. Moreover, studies have linked nicotine dependence to mental health disorders, including depression and bipolar disorder, where nicotine initially provides mood stabilization but ultimately exacerbates mood swings and depressive episodes^19,20^. This highlights the need for treatment approaches that address both nicotine dependence and underlying psychological conditions.

Given the established connection between anhedonia and nicotine use, this study examines how this relationship manifests among female college students. It assesses the prevalence of anhedonic symptoms and their association with nicotine use, providing insights into how these factors intersect in this population. The findings will contribute to targeted interventions aimed at reducing nicotine use rates and improving mental health outcomes among female undergraduate students.

## METHODS

### Study design

This study employed a cross-sectional design to investigate the association between anhedonia and nicotine dependence among female undergraduate students in Saudi Arabia.

### Study population and sampling

This study focuses on female undergraduate students as gender-specific factors shape health behaviors and stress levels^21^. Examining female students independently allows for a more precise understanding of the factors influencing their nicotine use and mental health.

In addition, this study aims to explore patterns within young females rather than compare genders. Previous studies have effectively examined female health behaviors without requiring a male comparison group^22^, demonstrating that meaningful insights can be drawn from single-gender studies. Furthermore, focusing on female participants ensures that the findings remain directly applicable to interventions and policies tailored to their health in similar educational and social settings.

A sample of 449 students was selected using a non-probability convenience sampling technique. The total undergraduate student population was 26500. Based on Raosoft software calculations, a minimum sample size of 380 students was required to achieve a 95% confidence level with a 5% margin of error.

### Instruments

*Questionnaire*

The study utilized a 29-item questionnaire, which included 9 items on demographic characteristics, mental health conditions, substance use other than nicotine, and nicotine use in immediate family members, as well as nicotine use and nicotine products. The questionnaire also incorporated 6 items from the Fagerström test for nicotine dependence (FTND) and 14 items from the Snaith-Hamilton Pleasure Scale (SHAPS)^23,24^.

Mental health conditions were assessed by asking participants: ‘Have you ever been diagnosed with a mental health condition by a healthcare professional?’ (yes/no), without specifying conditions to ensure privacy and encourage honest reporting. Substance use was similarly assessed by asking: ‘Do you currently use any substances other than nicotine?’ (yes/no).

Nicotine use in immediate family members (parents and siblings) was assessed by asking: ‘Do any of your parents use nicotine products?’ (yes/no) and ‘Do any of your siblings use nicotine products?’ (yes/no).

Nicotine use was assessed with the question: ‘Do you currently use any nicotine products?’ (yes/no). Participants who answered ‘yes’ were directed to specify the nicotine product they use and to complete the FTND items to assess nicotine dependence, followed by the SHAPS. Participants who answered ‘no’ were directed only to the SHAPS.

*Fagerström test for nicotine dependence (FTND)*

The FTND consists of six questions designed to assess the level of nicotine dependence. The questions are scored to categorize the level of nicotine dependence. Total scores range from 1 to 10, interpreted as: 1–2=low dependence, 3–4=low to moderate dependence, 5–7=moderate dependence, and ≥8=high dependence^23^.

*Snaith-Hamilton Pleasure Scale (SHAPS)*

The SHAPS is a validated tool for assessing anhedonia. It consists of 14 items, each scored as 0 or 1. Total scores range from 0 to 14, with scores >2 indicating anhedonia^24^.

The FTND and SHAPS have demonstrated strong psychometric properties, ensuring their reliability and validity in both clinical and research settings. The FTND shows moderate to good test-retest reliability, with scores ranging from 0.56 to 0.92 across various populations, including adolescents, psychiatric groups, and the general population. In smokers without psychotic symptoms, the FTND exhibited higher reliability (0.82) compared to other smoking-related scales^25^. The SHAPS has been validated as a reliable, unidimensional instrument for assessing hedonic capacity in adult outpatients with Major Depressive Disorder, showing a statistically significant correlation with health-related quality of life and functional impairment^26^.

### Ethical considerations

The data collection process began in March 2024, following authorization from the administrative department and approval from the Institutional Review Board of Princess Nourah bint Abdulrahman University (IRB Number: 24-0517; Date: 18 February 2024). Students were provided with a self-administered quantitative questionnaire, which was distributed via Google Forms, email, and WhatsApp. To ensure students could complete the surveys independently, the decision was made not to administer them directly. The study was conducted in accordance with the Declaration of Helsinki.

### Statistical analysis

The analysis was conducted using IBM^®^ SPSS^®^ Statistics software, version 30. Cronbach’s alpha was used to assess the reliability of the FTND and SHAPS scales, yielding values of 0.71 and 0.83, respectively. Nicotine dependence and anhedonia scores were presented as median and interquartile range (IQR) due to their non-normal distribution, as determined by the Shapiro–Wilk test. A chi-squared test was used to compare categorical groups, and a binary logistic regression model was conducted to assess the association while controlling for potential confounders. A p<0.05 was considered statistically significant in the two-tailed analysis.

## RESULTS

### Demographic characteristics

A total of 449 female undergraduate students participated in the study, as shown in Supplementary file Table 1. Most participants were aged 18–20 years (62.8%), followed by those aged 21–23 years (32.5%) and ≥24 years (4.7%). Most students were in their first year of study (37.9%), with the rest distributed across the second to seventh years. Only 11.4% of students reported nicotine use.

**Table 1:** Comparisons between nicotine users and non-users, among female students, Saudi Arabia 2024

*Variable*	*Categories*	*Non-users* *(N=398)*		*Nicotine users* *(N=51)*		*χ*^2^ *(df)*	*p*
		*n*	*%*	*n*	*%*		
**Parental nicotine use**	Yes	105	26.4	23	45.1	7.77 (1)	0.005
	No	293	73.6	28	54.9		
**Sibling nicotine use**	Yes	122	30.7	27	52.9	10.13 (1)	0.001
	No	276	69.3	24	47.1		
**Substance use (non-nicotine)**	Yes	35	8.8	4	7.8	0.05 (1)	0.820
	No	363	91.2	47	92.2		
**Mental health conditions**	Yes	60	15.1	20	39.2	17.99 (1)	<0.001
	No	338	84.9	31	60.8		

### Nicotine use

Overall, 11.4% (51/398) of participants reported nicotine use, with electronic cigarettes being the most commonly used product (66.7%), followed by traditional cigarettes (25.5%) and hookah (7.8%). Nicotine use was more prevalent among the immediate family members of nicotine users than non-users (Table 1). Specifically, 45.1% of nicotine users reported parental nicotine use, compared to 26.4% of non-users [χ^2^(1)=7.770, p=0.005], while sibling nicotine use was reported by 52.9% of nicotine users versus 30.7% of non-users [χ^2^(1)=10.128, p=0.001]. These findings highlight a potential familial influence on nicotine use patterns among participants.

Substance use was relatively low, reported by 8.7% of all participants. There was no significant difference in substance use between non-users (8.8%) and nicotine users (7.8%) [χ^2^(1)=0.052, p=0.820]. Mental health conditions were reported by 17.8% of participants, with a significantly higher prevalence among nicotine users (39.2%) compared to non-users (15.1%) [χ^2^(1)=17.992, p<0.001] (Table 1).

### Regression analyses of nicotine use factors

A binary logistic regression analysis was performed to investigate the influence of immediate family members’ nicotine use, mental health conditions and substance use on nicotine use.

The model was statistically significant [χ^2^(4)=32.344, p<0.001], explained 14% of the variance in nicotine use and correctly classified 88.6% of the participants. Parental nicotine use (p=0.01), sibling nicotine use (p=0.006) and mental health conditions (p<0.001) were significant predictors with each unit increase leading to 2.27, 2.37 and 4.44 times increase in the odds, respectively (Table 2). These findings indicate that parental nicotine use, sibling nicotine use and mental health conditions are important factors in determining the likelihood of nicotine use with mental health conditions being the most influential predictor.

**Table 2:** Factors associated with nicotine use within a binary logistic regression analysis among female students, Saudi Arabia 2024

*Variable*	*B*	*SE*	*Wald*	*p*	*OR*	*95% CI*	
						*Lower*	*Upper*
**Parental nicotine use**	0.82	0.32	6.43	0.011	2.27	0.48	1.49
**Sibling nicotine use**	0.86	0.32	7.49	0.006	2.37	1.15	3.23
**Substance use (non-nicotine)**	-0.64	0.60	1.12	0.290	0.53	0.71	3.46
**Mental health conditions**	1.492	0.343	18.90	<0.001	4.444	1.96	6.14

Dependent variable: nicotine use. Nicotine users set as the dummy variable (value=1), with non-users as the reference (value=0). B: unstandardized β. SE: standard error.

### Nicotine dependence

Responses to the FTND indicated a median nicotine dependence score of 8.00 (IQR: 6.00–9.00) out of 10. Participants were categorized as follows: low dependence (median=2.00, IQR=2–2, n=2; 3.9%), low to moderate dependence (median=3.5, IQR=3–4, n=6; 11.8%), moderate dependence (median=6, IQR=6–7, n=15; 29.4%), and high dependence (median=9, IQR=8–10, n=28; 54.9%). Due to the small sample sizes in the first three categories, they were combined into a single ‘low nicotine dependence’ group (median=6, IQR=4–6, n=23; 45.1%) to enhance statistical reliability.

As shown in Table 3, chi-squared analysis revealed that parental nicotine use was marginally associated with nicotine dependence, with a higher prevalence in the high-dependence group (57.1%) compared to the low-dependence group (30.4%) [χ^2^(1)=3.638, p=0.056]. No significant differences were found between the groups in terms of nicotine product, sibling nicotine use, substance use, or mental health conditions.

**Table 3:** Comparisons between low (N=23) and high nicotine dependence groups (N=28) among female students, Saudi Arabia 2024

*Variable*	*Categories*	*Low nicotine dependence*		*High nicotine dependence*		*χ*^2^ *(df)*	*p*
		*n*	*%*	*n*	*%*		
**Nicotine product**	E-cigarettes	14	60.9	20	71.4	0.63 (1)	0.43
	Other	9	39.1	8	28.6		
**Parental nicotine use**	Yes	7	30.4	16	57.1	3.64 (1)	0.056
	No	16	69.6	12	42.9		
**Sibling nicotine use**	Yes	10	43.5	17	60.7	1.51 (1)	0.22
	No	13	56.5	11	39.3		
**Substance use (non-nicotine)**	Yes	2	8.7	2	7.1	0.04 (1)	0.84
	No	21	91.3	26	92.9		
**Mental health conditions**	Yes	9	39.1	11	39.3	0.00 (1)	0.991
	No	14	60.9	17	60.7		

### Anhedonia

The anhedonia scores among participants were relatively low, with a median of 1.00 (IQR: 0.00–2.00) out of 14. Most participants were categorized as non-anhedonic (≤2), median 1.00 (IQR: 0.00–2.00), n=360 (80.2%), while 89 (19.8%) participants were categorized as anhedonic (>2), with a median score of 5.50 (IQR: 4.00–7.50). These findings indicate a relatively low prevalence of anhedonia in this population.

*Associations with nicotine use, substance use, and mental health*

Mental health conditions were significantly more prevalent among anhedonic participants (38.2%) compared to non-anhedonic participants (12.8%) [χ^2^(1)=31.501, p<0.001]. Nicotine use was also significantly more common among anhedonic participants (27%) than among non-anhedonic participants (7.5%) [χ^2^(1)=8.309, p=0.005]. Similarly, sibling nicotine use was significantly higher in anhedonic participants (46.1%) compared to non-anhedonic participants (30%) [χ^2^(1)=26.857, p<0.001]. However, no significant associations were found between anhedonia and nicotine product, nicotine dependence and parental nicotine use (Table 4).

**Table 4:** Comparisons among anhedonia groups among female students, Saudi Arabia 2024

*Variable*	*Categories*	*Non-Anhedonic*		*Anhedonic*		*χ*^2^ *(df)*	*p*
		*n*	*%*	*n*	*%*		
**Nicotine use**	Yes	27	7.5	24	27	26.86 (1)	<0.001
	No	333	92.5	65	73		
**Nicotine product**	E-cigarettes	21	77.8	13	54.2	3.19 (1)	0.074
	Other	6	22.2	11	45.8		
**Nicotine dependence**	High	15	55.6	13	54.2	0.01 (1)	0.921
	Low	12	44.4	11	45.8		
**Parental nicotine use**	Yes	102	28.3	26	29.2	0.027 (1)	0.869
	No	258	71.7	63	70.8		
**Sibling nicotine use**	Yes	108	30	41	46.1	8.31 (1)	0.004
	No	252	70	48	53.9		
**Substance use (non-nicotine)**	Yes	27	7.5	12	13.5	3.22 (1)	0.073
	No	333	92.5	77	86.5		
**Mental health conditions**	Yes	46	12.8	34	38.2	31.50 (1)	<0.001
	No	314	87.2	55	61.8		

*Regression analyses of anhedonia factors*

A binary logistic regression analysis was performed to investigate the effect of nicotine use among participants and their immediate family members, mental health conditions and substance use on the likelihood of experiencing anhedonia. The model was statistically significant [χ^2^(5)=48.932, p<0.001], and explained 16.4% of the variance in anhedonia and correctly classified 79.7% of the participants. In the model, mental health conditions (p<0.001), nicotine use (p<0.001) and sibling nicotine use (p=0.013) were significant with each unit increase leading to 3.47, 3.34 and 1.92 times increase in the odds, respectively (Table 5).

**Table 5:** Factors associated with anhedonia within a binary logistic regression analysis among female students, Saudi Arabia 2024

*Variable*	*B*	*SE*	*Wald*	*p*	*OR*	*95% CI*	
						*Lower*	*Upper*
**Nicotine use**	1.21	0.33	12.21	<0.001	3.34	1.72	6.49
**Parental nicotine use**	-0.17	0.29	0.34	0.563	0.85	0.48	1.49
**Sibling nicotine use**	0.66	0.26	6.19	0.013	1.93	1.15	3.23
**Substance use (non-nicotine)**	0.45	0.40	1.24	0.265	1.57	0.71	3.46
**Mental health conditions**	1.24	0.29	18.30	<0.001	3.47	1.96	6.14

Dependent variable: Anhedonia. Anhedonic set as the dummy variable (value=1), with non-anhedonic as the reference (value=0). B: unstandardized β. SE: standard error.

## DISCUSSION

This study investigated the prevalence of anhedonia among female undergraduate students and explored its association with nicotine use and dependence. These findings indicate that mental health conditions, nicotine use, and sibling nicotine use are important factors in determining the likelihood of experiencing anhedonia, with mental health conditions being the most influential predictor, followed by nicotine use.

Anhedonia symptoms were reported by 19.8% of the participants, while nicotine use was reported by 11.4% of the participants, the majority used electronic cigarettes. Recent research highlights the distinct issues faced by young female students regarding nicotine use and mental health, especially as they cope with academic expectations and social influences^27,28^. Almutairi et al.^29^, found that young female students are increasingly utilizing alternative nicotine products, including electronic cigarettes as evident by the findings of the current study which indicates that 66.7% of nicotine users employed electronic cigarettes. This can be ascribed to the perceived decrease in health hazards linked to e-cigarettes relative to conventional smoking, coupled with its convenience and attractiveness due to flavors and discreet use^30-32^.

The findings suggested association between anhedonia and nicotine use, with anhedonic participants showing a higher prevalence of nicotine use. Mental health conditions and sibling nicotine use were also considered as potential factors associated with anhedonia. A binary logistic regression model indicated that nicotine use and mental health conditions had comparably strong influences on the likelihood of experiencing anhedonia. Anhedonia was identified as a substantial withdrawal symptom that can prolong smoking habit. Higher levels of post-quit anhedonia were linked to worse success rates in quitting smoking^8^. This suggests that women who experience diminished ability to feel pleasure may become more reliant on nicotine’s mood-enhancing effects. Indeed, young women with depressive or anhedonic vulnerability often report smoking as a form of mood regulation or relief from negative affect^33^, supporting the notion that psychological distress can drive nicotine use in this population. Studies by Kim et al.^8^ and Cook et al.^9^ have examined how anhedonia can impede smoking cessation, since persons with elevated anhedonia may find it challenging to derive satisfaction or pleasure independent of nicotine use. Additionally, a study by Conti et al.^10^, observed that young women exhibiting increased anhedonic symptoms may face a heightened risk of long-term nicotine dependence, underscoring the necessity of incorporating mental health considerations into rehabilitation strategies.

Nicotine dependence levels in our sample were generally low to moderate, which aligns with patterns observed in similar populations. Many university-age smokers tend to consume relatively few cigarettes, and recent surveys of college students report that most young smokers exhibit low nicotine dependence^34^. This lower dependence may reflect a social or intermittent smoking pattern typical of this age group, yet even light-to-moderate smoking can maintain dependence through conditioned rewards. The consistency of our results with other college samples suggests that while these young women are not heavily nicotine-dependent by clinical standards, they may still face challenges in quitting due to the reinforcing psychological effects of smoking (e.g. stress relief, social facilitation). Notably, the increased likelihood of depressive symptoms in women and their possible association with nicotine use have been documented^35^, implying that even low-level smokers could be self-medicating subtle mood disturbances. Thus, interventions for young female smokers should not overlook those with lower usage, as their dependence, albeit mild, is intertwined with emotional well-being.

Among nicotine users, 45% reported parental nicotine use, 52% reported sibling nicotine use, and 39% reported mental health conditions. These factors were significantly associated with nicotine use, with mental health being the most influential predictor according to the binary logistic regression model. These findings emphasize the profound impact of mental health and familial nicotine use on smoking behavior, which has been consistently highlighted in the literature. Andreasen^19^ noted that young women are particularly vulnerable to nicotine addiction when they have concurrent psychiatric disorders such as depression and anxiety. Furthermore, Elsary and El-Sherbiny^36^, found that nicotine use is often employed as a coping mechanism for psychological distress, highlighting a similar pattern of increased mental health issues among smokers. The higher prevalence of anhedonic symptoms observed in our study further supports the notion that nicotine may be used to manage emotional discomfort, reinforcing the need for integrated approaches that address both nicotine dependence and mental health challenges.

Adouani et al.^37^ highlighted that parental and sibling nicotine use practices are significant predictors of smoking beginning in young women. Steeger et al.^38^ also highlighted that familial smoking practices notably increase the risk of smoking initiation among adolescents. These findings emphasize the importance of targeting the family environment in smoking prevention initiatives, particularly for young female students, as addressing familial smoking behaviors can be a crucial component of effective smoking cessation and prevention programs.

The results of this study contribute to ongoing research on nicotine dependence among young women, revealing a complex relationship with mental health, and familial nicotine use. The findings highlight how these variables interconnect, underscoring the need for tailored prevention and cessation programs that address the unique contexts of female undergraduate students. Specifically, the study points to anhedonia as a significant psychiatric condition that may perpetuate nicotine dependence. To effectively tackle this issue, prevention and cessation programs must integrate mental health treatment, focusing on addressing underlying psychological challenges such as anhedonia, to support more comprehensive and lasting recovery from nicotine dependence.

### Limitations

Although this study highlights the association between anhedonia and nicotine use, it was not possible to establish causality due to the study’s cross-sectional design. Also, as our sample was not randomly selected, the findings have limited generalizability to all female undergraduate students in Saudi Arabia and beyond. In addition, the small number of nicotine users reduces statistical power, making it more difficult to draw reliable conclusions. Self-reported smoking behavior and mental health issues may be influenced by underreporting, social desirability bias, and recall bias. Moreover, although students who reported nicotine use were required to complete the FTND before the SHAPS to ensure their responses to relevant assessments, the use of the FTND, designed specifically for cigarette users, is another limitation of this study. Furthermore, there might be several unmeasured factors that have influenced the results. For example, personality traits have been shown to play a role in both smoking behavior and anhedonia^39^. To enhance the findings of the present study, future research should employ longitudinal designs, incorporate data from a broader range of groups, and adapt the FTND or use alternative measures appropriate for different nicotine products.

## CONCLUSIONS

This study offers significant insights into the association between nicotine use and anhedonia in female undergraduate students. Despite the relatively low nicotine use prevalence, this research indicates that electronic cigarette usage is prevalent among young female nicotine users. This finding signifies a shift in nicotine use trends, aligning with prior research highlighting the increasing incidence of electronic cigarette use. The study’s results support the notion that psychological discomfort influences nicotine use, demonstrating a substantial association between anhedonia and nicotine use. Given the influence of familial nicotine use, there is an immediate need for targeted interventions that address both social and psychological aspects.

## Supplementary Material



## Data Availability

The raw data supporting the conclusions of this article will be made available by the authors, without undue reservation.
