# Supplementary material for: Exploring the association between anhedonia and nicotine dependence: A study among female undergraduate students in Saudi Arabia
Source: Tob Induc Dis. 2025 Apr 30;23:10.18332/tid/203551. doi: 10.18332/tid/203551 (PMC12042275; doi:10.18332/tid/203551)
Supplement: Supplementary file 1 [file TID-23-52-s1.pdf]

Supplementary Table 1: Demographic characteristics.

Variable	Category	n.	%
Age group	18-20	282	62.8
	21-23	146	32.5
	24+	21	4.7
Year of university studies	First	170	37.9
	Second	104	23.2
	Third	58	12.9
	Fourth	81	18.0
	Fifth	11	2.4
	Sixth	13	2.9
	Seventh	12	2.7
Nicotine use	Yes	51	11.4
	No	398	88.6

© 2025 Alwesmi M.B. et al.
